# Understanding Food Insecurity as a Determinant of Health in Pregnancy Within the United States: An Integrative Review

**DOI:** 10.1089/heq.2023.0116

**Published:** 2024-03-21

**Authors:** Veronica C. Pasha, Lauren Gerchow, Audrey Lyndon, Maya Clark-Cutaia, Fay Wright

**Affiliations:** Department of Nursing, New York University Rory Meyers College of Nursing, New York, New York, USA.

**Keywords:** food insecurity, pregnancy outcomes, maternal inequities, social and structural determinants of health

## Abstract

**Background:**

Food insecurity is a major public health concern in the United States, particularly for pregnant and postpartum individuals. In 2020, ∼13.8 million (10.5%) U.S. households experienced food insecurity. However, the association between food security and pregnancy outcomes in the United States is poorly understood.

**Purpose:**

The purpose of this review was to critically appraise the state of the evidence related to food insecurity as a determinant of health within the context of pregnancy in the United States. We also explored the relationship between food insecurity and pregnancy outcomes.

**Methods:**

PubMed, CINAHL, Web of Science, and Food and Nutrition Science databases were used. The inclusion criteria were peer-reviewed studies about food (in)security, position articles from professional organizations, and policy articles about pregnancy outcomes and breastfeeding practices. Studies conducted outside of the United States and those without an adequate definition of food (in)security were excluded. Neonatal health outcomes were also excluded. Included articles were critically appraised with the STROBE and Critical Appraisal Skills Program checklists.

**Results:**

Nineteen studies met the inclusion criteria. Inconsistencies exist in defining and measuring household food (in)security. Pregnant and postpartum people experienced several adverse physiological and psychological outcomes that impact pregnancy compared with those who do not. Intersections between neighborhood conditions and other economic hardships were identified. Findings regarding the impact of food insecurity on breastfeeding behaviors were mixed, but generally food insecurity was not associated with poor breastfeeding outcomes in adjusted models.

**Conclusion:**

Inconsistencies in definitions and measures of food security limit definitive conclusions. There is a need for standardizing definitions and measures of food insecurity, as well as a heightened awareness and policy change to alleviate experiences of food insecurity.

## Introduction

In 2020, 10.5% (∼13.8 million) of households in the United States experienced food insecurity.^1^ Of the households with children, the majority are headed by single mothers.^2^ Food insecurity, an economic and social condition characterized by the inability or limitation to access adequate food,^3^ is particularly important for women of reproductive age. Poor nutritional status can coexist with chronic health conditions, such as cardiovascular disease^4^ and type 2 diabetes mellitus,^5^ which put individuals at increased risk for obstetrical complications contributing to morbidity and mortality during pregnancy and within the first year postpartum.^6^

Recognition of the impact of mutable upstream factors,^7^ commonly referred to as “social determinants of health” (SDOHs), has significantly increased over the past 30 years.^8^ Economic stability, for example, is an SDOH characterized as the need to help people earn a living wage^9^ to carry out basic health needs, which includes the ability to acquire healthy foods. Concerns about accessibility and affordability of quality food in the United States have exacerbated during the COVID-19 pandemic, with the rate of U.S. households with children experiencing food insecurity increasing by 23%^10^ during the pandemic compared with the 2018 data.^11^

### Nutrition in pregnancy

Nutritional status of individuals before pregnancy is an important contributor to health during pregnancy and the lifecourse.^12^ For example, preconception bodyweight (both low and high) is associated with poorer pregnancy outcomes.^12^ Oftentimes, pregnancy is described as a “critical window” in women's health,^13^ as pregnant individuals are more inclined to adopt behavioral changes.^13^ However, nearly 50% of all pregnancies are unplanned.^14,15^ Therefore, nutritional education and access to preconception services for women of reproductive age are crucial to setting a foundation for a healthy pregnancy.^12^

Maintaining adequate nutritional status is vital for maternal well-being.^16^ Pregnant people are expected to gain a prescribed amount of weight, based on their prepregnancy body mass index (BMI), and return to an “appropriate” BMI through lifestyle behaviors^17^ within the first year postpartum. However, behavioral interventions largely place responsibility on the individual and do not address environmental factors, such as access to high-quality and nutritious foods,^18^ that affect capacity for behavior change. Most recommendations from the Dietary Guidelines for Americans pertain to the development of the fetus, maternal weight, and lactation goals. There is a gap in the guidelines for postpartum people who are not lactating and for those who may have experienced a metabolic complication in pregnancy, such as gestational diabetes mellitus (GDM).

## Purpose

There is a paucity of literature that examines the association between food security and pregnancy outcomes in the United States. The purpose of this integrative review is to (1) critically review the state of the evidence related to food insecurity as a determinant of health within the context of pregnancy in the United States and (2) describe the association of food insecurity as a determinant of pregnancy outcomes. Acknowledging that not all pregnant people will identify as women or mothers, gendered and nongendered terms will be used interchangeably. For context, ∼5.6% of adults in the United States identify as transgender,^19^ a subset of whom are transgender individuals of childbearing age.

## Methods

### Design

This integrative review was guided by the methodologies described by Toronto and Remington.^20^ To capture the multidisciplinary nature of this topic, literature searches were conducted in the PubMed, CINAHL, Web of Science, and Food and Nutrition Science databases. A health services librarian was consulted to increase specificity of studies.^20^ Searches took place between March and October 2022. Additional databases and search engines were manually searched to identify potential gray literature and minimize publication bias.^20^ Table 1 describes the search terms used.

**Table 1.** Literature search by database

Database	Search strategy
PubMed	(“Food Security”[Mesh] OR “food insecur^*^”[tw] OR “food secur^*^”[tw] OR “food desert^*^”[tw]) AND (“Perinatal Care”[Mesh] OR “Pregnancy”[Mesh]” OR “pregnan^*^”[tw])
CINAHL	((MH “Food Security”) OR (MH “Food Deserts”) OR (MH “Food Assistance”)) OR (“food security” OR “food insecur^*^” OR (food N3 access^*^) OR “food desert^*^”) AND ((MH “Maternal-Child Health”) OR (MH “Maternal Health Services+”)) OR (maternal OR pregnan^*^ OR “pre natal” OR prenatal OR perinatal OR postnatal OR postpartum) OR (MH “Pregnancy+”) OR (MH “Pregnancy Trimesters+”)
Web of Science	“food security” OR “food insecurity” OR “food deserts” OR “access NEAR/3 food” AND “pregnancy” OR “perinatal” OR “maternal health services OR pregnancy outcomes OR postpartum”
Food and Nutrition Sciences	Food security and (pregnancy or postpartum)

### Inclusion and exclusion criteria

The inclusion criteria included research articles published after 2006 and conducted within the United States about food security or insecurity written in English that were peer-reviewed; position articles from professional organizations; or policy articles with a discussion of pregnancy outcomes and breastfeeding practices. Articles that did not contain an adequate explanation or definition of food security or insecurity in relation to pregnancy were excluded. Conference abstracts, editorials, and publications that focused on neonatal health outcomes were also excluded.

### Search results

From the initial literature search, 2647 articles were imported into Covidence Systematic Review Software. Eight hundred eighty-two total duplicates were removed, yielding 1765 unique citations for title and abstract screening. Using strict inclusion and exclusion criteria, 147 full-text studies were assessed. Following a full-text review, 19 articles remained and were included for final analysis. Figure 1 presents the search strategy via Preferred Reporting Items for Systematic Reviews and Meta-Analyses (PRISMA).

**Figure:**
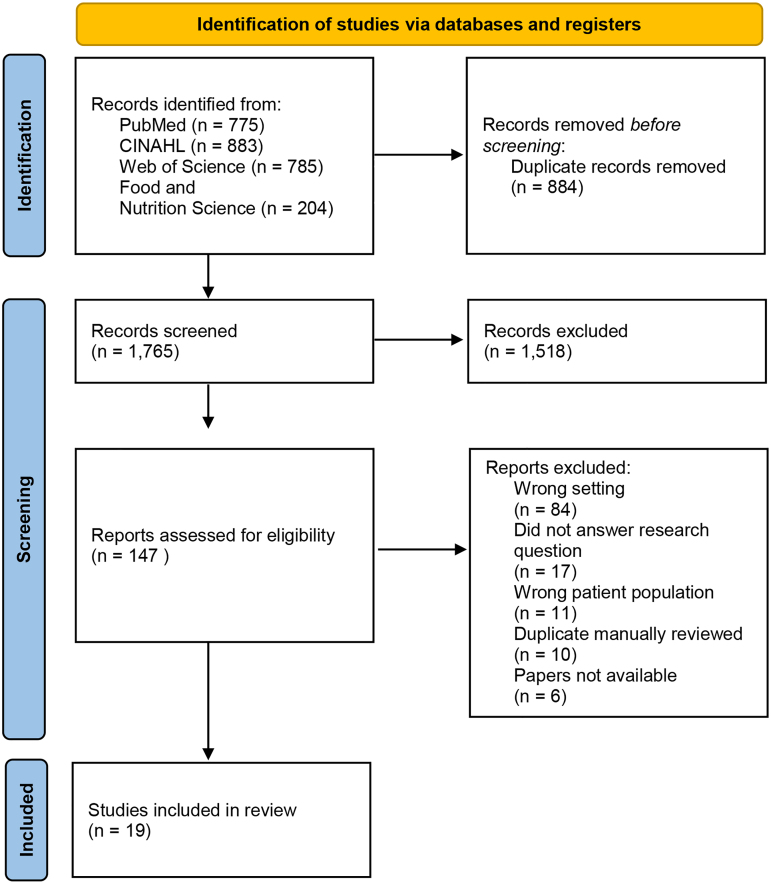
**FIG. 1.** This diagram demonstrates the systematic process as described by the Preferred Reporting Items for Systematic Reviews and Meta-Analyses (PRISMA) for included studies.

### Data analysis

Data analysis for this review used the constant comparison method.^21^ Studies were read in chronological order based on publication date. Data extraction was independently conducted by coauthors (V.C.P. and L.G.) using a data matrix. Codes were compared and then regrouped into separate columns with subheadings that described the factors and outcomes associated with food insecurity (Table 2).

**Table 2.** Synthesis matrix

Source	Psychological consequences				Physiological abnormalities					Maternal hardships
	Depression	Anxiety	Stress	Self-esteem and coping	Altered eating patterns and behaviors	Gestational weight gain		Pregnancy complications	Breastfeeding implications	Social inequities/disparities
						Excessive	Insufficient			
Laraia et al.^2^	X	X	X	X				X		X
Laraia et al.^29^						X		X		
Stevens^39^		X	X		X					X
Hromi-Fiedler et al.^37^			X	X						
Gross et al.^32^.									X	
Laraia et al.)^27^.					X	X				
Park and Eicher-Miller^35^								X		
Laraia et al.^28^			X		X	X				
Grilo et al.^24^	X									
Morales et al.^30^								X		X
Gross et al.^40^									X	X
Cheu et al.^25^			X				X			X
Dinour et al.^34^									X	
Orozco et al.^38^									X	
Frazier et al.^33^									X	
Sullivan et al.^23^				X	X					X
Sandoval et al.^31^		X	X							X
Cooper et al.^26^								X		
Laraia et al.^36^	X									X

### Critical appraisal

The Strengthening the Reporting of Observational Studies in Epidemiology (STROBE) checklist was used for cross-sectional and cohort studies. The Critical Appraisal Skills Program (CASP) checklist was used to appraise the qualitative studies, supplemented by triangulation (inclusion of multiple data points, sources, and researcher points of view) and reflexivity (awareness of and authenticity about one's own identity, role, and influence within the research context) as defined by Tracy and Hinrichs^22^ that are not mentioned in the CASP tool. Table 3 displays strengths and weaknesses of each study using the respective critical appraisal tools.

**Table 3.** Critical appraisal

Author (year) Appraisal tool	Study design	Strengths	Weaknesses
Cheu et al. (2020)^25^ *STROBE*	Cohort study	● Clear objectives and hypotheses ● Large diverse patient population ● Primary and secondary outcomes clearly stated ● Appropriate statistical analyses with confounders	● Possibility of selection bias due to one researcher selecting participants based on eligibility ● Social desirability bias ● Single site and may not be generalizable
Cooper et al. (2022)^26^ *STROBE*	Prospective cohort study	● Innovation and uniqueness of study are clearly detailed. ● Methods section is written clearly (although missing some details)	● Study reports projected number of participants without explanation (no power analysis) ● Regression modeling does not account for confounders. ● Results show wide confidence intervals without explanation ● No age mention for inclusion/exclusion criteria so unable to assess for ethical concerns
Dinour et al. (2020)^34^ *STROBE*	Cross-sectional	● Clearly defined potential confounders and moderators ● Clear rationale for statistical methods ● Descriptive data well displayed and explained	● Minimal discussions of bias ● Single-item question to determine food insecurity status without psychometrics reported
Frazier et al. (2021)^33^ *STROBE*	Cross-sectional	● Results section is written clearly and concisely. ● Background section clearly explains importance for exploration	● Modification of USDA module without explanation of question selection or reported psychometrics (No. of items and time frame) ● No clear classification of food security status ● Unclear if the study is generalizable ● No explanation of study size (which is small) ● Statistical analysis unclear related to that type of “exact” tests
Grilo et al. (2015)^24^ *STROBE*	Cohort study	● Population of interest clearly explained ● Survey measures with appropriate psychometrics are stated and well supported with reliability and validity scores. ● Well-explained rationale for categorization of food security	● Although population of interest is made clear, risk factors for this population as to “why” are underdeveloped. ● Ethical considerations for adolescent group
Gross et al. (2012)^32^ *STROBE*	Cross-sectional	● Excellent description of variables and potential confounders ● Results section is organized and answers research question ● Accurate reporting of statistical findings (with adjusted and unadjusted values) ● Use of path analysis	● No reported limitations ● Difficult to give interpretation of study without limitations and generalizability of study.
Gross et al. (2019)^40^ *CASP* Eight “Big-Tent” Criteria	Qualitative	● Clear methodology section and appropriate interpretation of thematic analysis. ● Interview guide was multidisciplinary, multicultural collaboration for diverse population of interest ● Clear delineation of roles within the research team	● No ethical considerations noted ● Limited generalizability ● Missing information on inclusion/exclusion criteria
Hromi-Fiedler et al. (2011)^37^ *STROBE*	Cross-sectional	● Multidisciplinary approach provides a more encompassing worldview of the stated problem ● Correlation matrices are accurate, with appropriate contingency statistical analyses used. ● Reported unadjusted and adjusted OR for precision ● Excellent distinction of Hispanic/Latinx group and mention of acculturation (understudied phenomenon)	● No clear explanation on how adaptation to survey was made ● Claimed enough statistical power but there is no mention of power analysis or effect size ● Inclusion/exclusion criteria could have had more information
Laraia et al. (2006)^2^ *STROBE*	Cross-sectional	● Population is clearly defined, and inclusion criteria follow throughout ● Appropriate surveys were selected to answer the research question ● Selected confounders are well supported with literature ● Findings were consistent for the general population	● Not all survey measures listed reliability psychometrics ● Regression models were run separately due to “high coefficients,” this increases the chance of a type I error ● *p*-Values set at 0.03 and 0.008 with no explanation for such ● No mention of bias
Laraia et al. (2010)^29^ *STROBE*	Secondary analysis of prospective cohort study	● Clear explanation of trained research staff conducting data abstraction from EMR ● Pregnancy outcome measures clearly defined and supported	● Inclusion and exclusion criteria for cohort not clearly stated. Only mention is restriction of household income, but overall N differs from previous study using same data. ● Subjective and self-reported data could lead to social desirability bias ● Inaccurate use and explanation of OR ● Minimal explanation for missing data
Laraia et al. (2013)^27^ *STROBE*	Secondary analysis of prospective cohort study	● Clear inclusion and exclusion criteria compared with previous studies ● Appropriate use, display, and explanation of statistical tests	● Inclusion and exclusion criteria for cohort not clearly stated. Only mention is restriction of household income ● Study uses same data set as previous publications, with similar design, and analyses therefore, I feel this leads to a bias study
Laraia et al. (2015)^28^ *STROBE*	Secondary analysis of prospective cohort study	● Population is clearly defined, and inclusion criteria follow throughout ● Appropriate surveys were selected to answer the research question ● Selected confounders are well supported with literature ● Findings were consistent for the general population	● Self-reported weight contributes to social desirability bias ● Sample size calculation was not justified and may contribute to committing a type I error
Laraia et al. (2022)^36^ *STROBE*	Secondary analysis of cross-sectional survey	● Key elements of research problem are indicated and supported ● Use of theoretical framework is threaded throughout article for clarity ● Statistical analyses are well explained and displayed in appropriate tables ● Study sample and missing data are well explained	● Chance of selection bias: participants with severe maternal hardship may be underrepresented ● Introduction mentions causal relationship, but this study did not execute that plan
Morales et al. (2016)^30^ *STROBE*	Retrospective cohort study	● Clearly stated desire to determine beneficial or harmful effect ● Appropriate statistical tests and analyses ● Confounders stated and adjusted	● Sampling strategy is not clear/does not appear to be randomized ● There is no standardized tool to measure food security ● No ethical considerations ● No clear inclusion/exclusion criteria ● Use of propensity matching could have been further explained
Orozco et al. (2020)^38^ *STROBE*	Secondary analysis of cross-sectional data	● Clear inclusion/exclusion criteria ● Well supported covariates ● Clear explanations of exposure and outcome variables ● Appropriate explanation of multiple ethical considerations	● Respondents to NHANES were primarily males and may have lead to recall bias
Park and Eicher-Miller (2014)^35^ *STROBE*	Secondary analysis of cross-sectional Data	● Clear objectives and statement of problem ● Excellent statistical explanation (using log and link transformation for non-normal distribution) ● Adjusted OR appropriately explained ● Statistical findings supported appropriately in discussion	● 13.7% of trimester data are missing with no explanation to account for this ● There is no mention of limitation or biases ● Discussion on sample size calculation is missing
Sandoval et al. (2021)^31^ *STROBE*	Retrospective cohort study	● All variables and outcomes were clearly defined ● Measuring tools report appropriate psychometrics ● Robust discussion that answered original aim ● Limitations discussed in abundance (although with so many limitations, it seems to take away from the many strengths this article holds)	● No description of inclusion/exclusion criteria (minimal mention of how final sample size was obtained) ● Methods section lacks follow-up logistics ● Participant characteristics table was underdeveloped and may not be indicative of the population studied (i.e., missing age) ● Data analysis is missing information on final logistic model that was put together
Stevens (2010)^39^ *CASP* Eight “Big-Tent” Criteria	Qualitative	● Topic is significant, interesting, and relevant ● Validated survey	● Lacks rigor due to minimal sincerity or triangulation ● No ethical consideration ● No clear methodology, display of results sounds like a mixed-methods study design ● No reflexivity or mention of how analysis was conducted
Sullivan et al. (2021)^23^ *STROBE*	Prospective cohort study	● Clearly defined operational definition of food insecurity ● Clearly focused issue of postpartum women experiencing food insecurity. ● Use of validated survey to assess for food insecurity ● Appropriate statistical test used	● Possible selection bias ● Cohort was not representative of nationally defined population of people with food insecurity ● Subjective information collected that may lead to self-report bias ● No ethical considerations mentioned ● Poor statistical reporting (lack of possible confounding variables)

CASP, Critical Appraisal Skills Program; NHANES, National Health and Nutrition Examination Survey; USDA, United States Department of Agriculture.

## Results

### Article characteristics

The 19 articles included in this review were primarily quantitative, consisting of 9 cohort studies^23–31^ and 8 cross-sectional studies.^2,32–38^ Two qualitative studies were included.^39,40^

Multiple definitions and measurement tools to assess household food security were identified. Eight studies^2,25,27–29,31,35,37^ utilized the United States Department of Agriculture (USDA) definition of food security.^41^ Four studies described food insecurity from previous works.^23,24,36,39^ Only one study^2^ used the original U.S. Household Food Security Survey (USFSS), an 18-item survey used largely in adult, nonpregnant populations. Modifications to the scale were used in 10 of the 19 articles.^23,25,27–29,31,35–37,39^ Grilo et al.^24^ used a single question taken from the “Pregnancy Nutrition Questionnaire” that was developed by the California Health and Human Services Agency, which asks, “Do you ever run out of money or food stamps to buy food?” (p. 3, 2022). A “standardized assessment form” (p. 3) was used by Morales et al.^30^ Table 4 displays the original 18-item survey and demonstrates which items remained through modifications.

**Table 4.** United States Department of Agriculture food security survey(s)

Survey items	USFSS module (18-items)	U.S. Adult Food Security Survey module (10-items)	USDA six-item, short form	Dinour et al. (2020)^34^
Household questions				
“(I/We) worried whether (my/our) food would run out before (I/we) got money to buy more.” *in the last 12 months*	X	X		
“The food that (I/we) bought just didn't last, and (I/we) didn't have money to get more.”	X	X	X	
“(I/we) couldn't afford to eat balanced meals.”	X	X	X	
Adult stage				
In the last 12 months, since last (name of current month), did (you/you or other adults in your household) ever cut the size of your meals or skip meals because there wasn't enough money for food?	X	X	X	
*[IF YES ABOVE, ASK] How often did this happen—almost every month, some months but not every month, or in only 1 or 2 months?*	X	X	X	
In the last 12 months, did you ever eat less than you felt you should because there wasn't enough money for food?	X	X	X	X
In the last 12 months, were you every hungry but didn't eat because there wasn't enough money for food?	X	X	X	
In the last 12 months, did you lose weight because there wasn't enough money for food?	X	X		
Adult stage 3				
In the last 12 months, did (you/you or other adults in your household) ever not eat for a whole day because there wasn't enough money for food?	X	X		
How often did this happen—almost every month, some months but not every month, or in only 1 or 2 months?	X	X		
Child screener				
“(I/we) relied on only a few kinds of low-cost food to feed (my/our) child/the children) because (I was/we were) running out of money to buy food.” Was that often, sometimes, or never true for (you/your household) in the last 12 months?	X			
“(I/We) couldn't feed (my/our) child/the children) a balanced meal, because (I/we) couldn't afford that.”	X			
“(My/our child was/the children were) not eating enough because (I/we) just couldn't afford enough food.”	X			
Stage 2 child screener				
In the last 12 months, since (current month) of last year, did you ever cut the size of (your child's/any of the children's) meals because there wasn't enough money for food?	X			
In the last 12 months, did (CHILD'S NAME/any of the children) ever skip meals because there wasn't enough money for food?	X			
*How often did this happen—almost every month, some months but not every month, or in only 1 or 2 months?*	X			
In the last 12 months (was your child/were the children) ever hungry but you just couldn't afford more food?	X			
In the last 12 months, did (your child/any of the children) ever not eat for a whole day because there wasn't enough money for food?	X			

USFSS, U.S. Household Food Security Survey.

Sample populations within each study were reflective of the U.S. pregnant population. The samples were diverse in age, race, ethnicity, income, education status, and public and/or private insurance. Included studies were reflective of most geographic regions in the United States. One article specifically described rural communities.^23^ Four articles were conducted in urban settings.^24,25,31,37^ Urbanicity was unable to be identified in eight studies as Laraia et al.,^2,27–29,36^ Dinour et al.,^34^ and Stevens^39^ used state-specific data. Orozco et al.^38^ and Park and Eicher-Miller^35^ used national data. Research aims in the included studies focused on describing the predictors and prevalence of food insecurity during pregnancy and investigating food insecurity as the predictive variable on several pregnancy complications, such as GDM,^26,29^ hypertension,^29,30^ and anemia.^35^ Table 5 provides a summary of the characteristics of the included studies.

**Table 5.** Summary of reviewed studies

Author (year) Geographic location	Aim	Methodology framework	Data collection Data analysis	Sampling strategy Sample	Household food insecurity Definition Tool Outcome	Major findings
Cheu et al. (2020)^25^ Location: Chicago, Illinois (Midwest)	“To examine the association between food insecurity and gestational weight gain in a diverse cohort of pregnant women” (p. 1).	Methodology: • Cohort design • No theoretical framework	Data collection: • Birth records within EMR • Inpatient survey • Modified version of the USDA Adult Food Security Survey Module Data analysis: • Mann–Whitney, Fisher exact, χ^2^, and *t*-tests • Multivariable multinomial logistic regression	Sampling strategy: • Purposive sampling Power analysis: α=0.05 and β=0.80 *n*∼237 Sample: • *n*=299 postpartum women • <18 years old • English speaking • Delivered live singleton >24 weeks gestation at a single tertiary care center	Definition: See Laraia et al.^2^ Measurement tool: Modified version of USDA Adult Food Security Survey Module Outcome: Categorical	• Participants with food insecurity are more likely to have sociodemographic characteristics of health inequities. Participants with food insecurity: • Lower total gestational weight gain *p*=0.009 • Hispanic/non-Hispanic black *p*<0.001 • Hold public insurance *p*<0.001 • Attended fewer PNC visits *p*<0.003 • Higher prepregnancy BMI *p*<0.001
Cooper et al. (2022)^26^ Location: Hartford, Connecticut (Northeast)	“To evaluate associations between food security and women diagnosed with gestational diabetes (GDM) and evaluate if women in food insecure households had adverse maternal and neonatal outcomes” (p. e131).	Methodology: • Prospective cohort study	Data collection: • Medical records • Inpatient Survey • Modified version of the USDA Adult Food Security Survey Module Data analysis: • Two-sided *t*-tests for continuous variables • Fisher's exact test for categorical variables • Logistic regression to test association	Sampling strategy: Convenience sampling from resident clinics contracted with UCONN Health system and Hartford Hospital Sample: • *n*=70 postpartum women (36 GDM; 34 NGDM) • English/Spanish speaking • Delivered live singleton >37 weeks gestation	Definition: “Access by all people at all times to enough good for an active, healthy life” (p. e131). Measurement tool: Modified version of USDA Adult Food Security Survey Module Outcome: Dichotomous	• Significantly higher rates of GDM in people with food insecurity (71%; *p*<0.001). Participants with food insecurity: • 50% identified as black • 46% identified as Hispanic • Unadjusted OR 5.02 (95% CI 1.77 to 15.32) • Adjusted OR 7.05 (95% CI 1.78 to 34.72)
Dinour et al. (2020)^34^ Location(s): Colorado, Maine, New Mexico, Oregon, Pennsylvania, and Vermont	“To utilize the national Pregnancy Risk Assessment Monitoring System (PRAMS) dataset to determine if food insecurity is associated with breastfeeding initiation and early cessation (<10 weeks) among U.S. mothers” (p. 3).	Methodology: • Secondary analysis of cross-sectional data	Data collection: • Public health survey • PRAMS ○ Birth certificates Data analysis: • χ^2^ tests, *t*-tests • Binomial logistic models (reported OR) • Multinomial logistic regressions (reported RR)	Sampling strategy: • Purposive sampling Sample: • *n*=10,159 • <20 years old • Delivered live neonate	Definition: “The lack of enough money to purchase adequate amounts, variety, and quality of food” (p. 2). Measurement tool: Single question from USDA 6-item food security module Outcome: Dichotomous	• Food insecurity was not associated with breastfeeding initiation after adjusting potential confounders
Frazier et al. (2021)^33^ Location(s): North Carolina (exact location unavailable)	“To examine breastfeeding initiation and continuation rates and investigate association between food insecurity and breastfeeding status, among low-income mothers” (p. 1).	Methodology: • Cross-sectional • No theoretical framework	Data collection: • Public health survey • PRAMS ○ Birth certificates Data analysis: • Descriptive statistics • χ^2^ and exact tests • Two-step hierarchal, binary logistic regression • Hosmer–Lemeshow test • Ordinary least-squares regression • Bootstrapping	Sampling strategy: • Convenience sampling from pediatric clinic Sample: • *n*=93 • >18 years old • Birth mother and main caretaker of infant • English or Spanish speaking • Singleton pregnancy born at >37 weeks gestation • Infants without health concerns or dietary restrictions • No income requirements, as participants were Medicaid recipients to participate in the clinic	Definition: “inconsistent access to a sufficient amount of high quality and nutritious food” (p. 2). Measurement tool: 10-item USDA Adult Household Food Security Survey Module (researchers modified for previous 30 days) Outcome: Dichotomous (assuming)	• Food insecurity not associated with discontinuation of breastfeeding at 2 months. • 80% participants (WIC) • 42% participants (SNAP)
Grilo et al. (2015)^24^ Location: New York, New York (Northeast)	“To document prevalence of food insecurity among pregnant adolescents, determine if food insecurity is associated with adverse birth outcomes, and examine whether depressive symptoms mediate these associations” (p. 2).	Methodology: • Cohort study of a previous RCT • No theoretical framework	Data collection: • Survey • Interviews at two time points Data analysis: • Descriptive statistics Path analysis	Sampling strategy: • Purposive Sample: • *n*=1233 • Pregnant adolescents (14–21 years old) • <24 weeks gestation • Low risk pregnancy • English or Spanish speaking	Definition: “Having limited or uncertain availability of nutritionally adequate food” (p. 1). Measurement tool: Pregnancy Nutrition Questionnaire developed by California Health and Human Services Agency Outcome: Categorical	• Acute and chronic food insecurity is associated with poor nutrition and greater depressive symptoms. Participants with food insecurity: • 24% reported depressive symptoms. • 29% reported mild anxiety. • Indirect effect of chronic food insecurity on birth weight via mediator of depressive symptoms β (SE)=−35.73 (15.69), *p*=0.02
Gross et al. (2012)^32^ Location: New York, New York (Northeast)	“Explored the relationship between household good insecurity and maternal feeding styles, infant feeding practices, and perceptions and attitudes about infant weight in low-income mothers” (p. 254).	Methodology: • Cross-sectional	Data collection: • Survey Data analysis: • Bivariate associations • Independent samples *t*-tests • One-way analysis of variance • Liner regression models • Logistic regression modeling	Sampling strategy: • Convenience • WIC center within large urban medical center Sample: • *n*=201 mother–infant dyads • >18 years old • Singleton delivery >37 weeks gestation • Infant age: 2 weeks–6 months • Infants with medical needs affecting feedings were excluded • English or Spanish speaking	Definition: “Limited or uncertain availability of nutritionally adequate and well-tolerated foods or the ability to acquire such foods” (p. 255). Measurement tool: Two questions from the CFSM Outcome: Categorical	There are no associations between food security status and breastfeeding patterns Participants with food insecurity: • More likely to show restrictive [β (SE)=0.18 (0.08); 95% CI: 0.02 to 0.34] and pressuring [β (SE)=0.11 (0.086); 95% CI: 0.001 to 0.22] feeding styles.
Gross et al. (2019)^40^ Location: New York, New York (Northeast)	“Gain a greater understanding of how food insecurity impacts feeding styles and practices” (p. 2).	Methodology: • Qualitative • Thematic analysis	Data collection: • Purposive • Mothers randomly assigned to the Starting Early Program intervention Data analysis: • Constant comparative methods • Coding	Sampling strategy: • Purposive • Interview guides designed by multicultural interdisciplinary team Sample: • *n*=100 • Self-identified Hispanic • <24 weeks gestation • All levels of food security (negative, marginal, positive) • English or Spanish speaking	Definition: “Having limited or uncertain availability of nutritionally adequate food” (p. 1). Measurement tool: CFSM Outcome: Categorical	Identified themes: • Contributors to general household financial strain and food insecurity • Effects of food insecurity on infant feeding • Coping strategies
Hromi-Fiedler et al. (2011)^37^ Location: Hartford, Connecticut (Northeast)	“To examine the independent association of household food insecurity with depressing symptoms among low-income pregnant Latinas” (p. 421).	Methodology: • Cross-sectional • No theoretical framework	Data collection: • Surveys • USFSS module Data analysis: • Bivariate correlations, χ^2^ and tests Stepwise regression modeling	Sampling strategy: • Convenience • From prospective study Sample: • >18 years old • Self-identified as Latina • 4–8 months pregnant • Reside in Hartford • Were not living in temporary housing	Definition: See Laraia et al.^2^ Measurement tool: Adapted from USFSS 15-item scale Cronbach α > 0.85 Outcome: Categorical	• Experiencing household food insecurity contributes to maternal stress. • 37% participants reported household insecurity (*n*=49) 40.8% (*n*=20) had elevated symptoms of depression *p*=0.05 • 31.6% reported their health during pregnancy as poor or fair (*n*=42) 54.8% (*n*=23) had elevated symptoms of depression *p*=0.000
Laraia et al. (2006)^2^ Location: North Carolina State (Southeast)	“To investigate the prevalence and predictors of food insecurity among pregnant women from medium- and low-income household” (p. 178).	Methodology: • Cohort study • No theoretical framework	Data collection: • Survey Data analysis: • Descriptive statistics χ^2^ and *t*-tests Logistic regression	Sampling strategy: • Convenience • From PIN Postpartum study Sample: • *n*=606 pregnant women • households <400% of the poverty line • >16-year olds • English speaking • Singleton pregnancy	Definition: “Whenever the availability of nutritionally adequate and safe food, or the ability to acquire acceptable foods in a socially acceptable ways, is limited or uncertain” (p. 177). Measurement tool: USDA 18-item scale Outcome: Categorical	Majority of participants experiencing food insecurity utilized coping strategies such as borrowing food, receiving food from a church or food bank, and sending their children to relatives to eat. Perceived stress independently associated with food insecurity • Adj OR 2.31 (1.27 to 2.68) Income as an independent predictor • χ^2^=32.25, *p*=0.001
Laraia et al. (2010)^29^ Location: North Carolina State (Southeast)	“To investigate the association between household food insecurity status and pre-gravid BMI, gestational weight gain, PIH, anemia, and GDM among pregnant women” (p. 3).	Methodology: • Cohort Study • No theoretical framework	Data collection: • Survey • EMR abstraction Data analysis: • One-way analysis of variance with Bonferroni • Multiple comparison tests • OR Linear regression modeling	Sampling strategy: • Convenience • From PIN postpartum study Sample: • *n*=810 pregnant women • households <400% of the poverty line • >16-year olds • English speaking • Singleton pregnancy	Definitions: See Laraia et al.^2^ Measurement tool: CFSM Outcome: Categorical	• Food insecure households were associated with severe obesity, second trimester anemia, and GDM. Food insecure (*n*=79) • PIH 38.0% (*n*=27), *p*<0.05 • Anemia 15.2% (*n*=16), *p*<0.05 • GDM OR 2.38 (0.99 to 5.73)
Laraia et al. (2013)^27^ Location: North Carolina State (Southeast)	“To test whether there may be an additive effect of being food insecure and exhibiting a high level of dietary restraint on gestational weight gain” (p. 3).	Methodology: • Cohort study • No theoretical framework	Data collection: • Survey between 26 and 31 weeks' gestation Data analysis: • χ^2^ and *t*-tests Linear regression models stratified by low and high dietary restraint	Sampling strategy: • Convenience • From PIN postpartum study Sample: • *n*=1041 pregnant women • >16-year-olds • English speaking • Singleton pregnancy	Definition: See Laraia et al.^2^ Measurement tool: CFSM Outcome: Dichotomous	• Exposure to food insecurity and high dietary restraint has a synergistic relationship while considering weight gain. • Significant interaction was found between marginal food insecurity and scoring high on dietary restraint with regard to gestational weight gain χ^2^=7.92, *p*<0.005
Laraia et al. (2015)^28^ Location: North Carolina State (Southeast)	“To investigate the influence of food insecurity on women's stress, disordered eating, dietary fat intake, and weight during the postpartum period” (p. 1303).	Methodology: • Cross sectional • No theoretical framework	Data collection: • Interviews • Chart review • Questionnaires • CFSM Data analysis: • Multivariate linear regression Effect modification	Sampling strategy: • Convenience • From PIN postpartum study Sample: • *n*=526 pregnant women • >16-year olds • English speaking • Singleton pregnancy	Definition: See Laraia et al.^2^ Measurement tool: CFSM Outcome: Categorical	• Experiencing food insecurity during pregnancy was associated with higher levels of stress and disordered eating. • Black women made up a large portion of food insecure group. Food security status during pregnancy • Perceived stress β=4.30 (2.12 to 6.49) • BMI β=1.74 (0.81 to 2.66) • EAT score β=1.95 (−0.25 to 4.16) Food security status postpartum • Perceived stress β=6.12 (3.86 to 8.38) • BMI β=0.93 (0.07 to 1.80) • EAT score β=1.79 (−0.03 to 3.62)
Laraia et al. (2022)^36^ Location: California State (West)	“To assess the extent to which severe maternal hardships are associated with food insecurity during pregnancy among a representative sample of low- and lower-income women who had live births in California” (p. 2).	Methodology: • Cross-sectional • Life Course Theory	Data collection: • Survey Data analysis: • χ^2^ tests to assess bivariate relationship • Multinomial logistic regression Stepwise gradient	Sampling strategy: • Random sampling, stratified on county/region of residence Sample: • *n*=14,274 postpartum women • >16-year olds • Delivered a live birth • Households <400% of the poverty line	Definition: “Multidimensional health risk that includes anxiety about, a lack of material resources for, and poor access to nutritious foods that can have implications across the lifecourse” (p. 1). Measurement tool: Modified version of USDA 6-item food security scale Outcome: Categorical	• Three out of four participants with food insecurity had at least one maternal hardship. Low food security • No practical support Adj RR 1.38 (1.02 to 1.87) Very low food security • Homelessness Adj RR 1.98 (1.08 to 3.63) • No emotional support Adj RR 1.64 (1.07 to 2.52)
Morales et al. (2016)^30^ Location: Chelsea, Massachusetts (Northeast)	“To determine whether participation in a food insecurity reduction program improved blood pressure and blood glucose levels in pregnant women” (p. 2).	Methodology: • Retrospective Cohort study • Propensity score matching • No theoretical framework	Data collection: • EMR extraction • If enrolled, data extracted from Food for Families. Data analysis: • χ^2^ tests for categorical variables • *t*-Tests for continuous variables • Linear mixed-effect models	Sampling strategy: • Purposive sampling Sample: • *n*=1295 pregnant women seen in obstetrics clinic • *n*=145 referred to Food for Families • <18 years old	Definition: “Limited or uncertain access to adequate food” (p. 1). Measurement tool: “Screened using standardized assessment form” Outcome: No mention of outcome measurements	• Intensity map showed most women seen at obstetrical clinic were food insecure, living below federal poverty level, Hispanic, and English as a second language. • During pregnancy, women enrolled in Food for Families had overall better SBP/DBP readings than those who were not. Referred to and enrolled in food for families • Improved SBP (0.2015 mm Hg/week lower) *p*=0.006 • Improved DBP (0.1049 mm Hg/week lower) *p*=0.02
Orozco et al. (2020)^38^ Location: United States, national data	“To examine the relationships among food insecurity, breastfeeding, and other related feeding practices by race/ethnicity among US infants and toddlers” (p. 588).	Methodology: • Secondary data analysis of cross sectional data	Data collection: • NHANES 2009–2014 Data analysis: • Survey-weighted, stratified multiple regression models	Sampling strategy: • Purposive sampling Sample: • *n*=2069 newborns and toddlers (58.5% <12 m)	Definition: “Limited or uncertain access to adequate food” (p. 1). Measurement tool: USDA 18-item Food Security Survey Module Outcome: Categorical	• 25% of study population was food insecure. • Food insecurity was higher among Hispanics • Food insecurity was not a significant predictor for breastfeeding initiation.
Park and Eicher-Miller (2014)^35^ Location: United States, national data	“To examine the hypothesis that food-insecure pregnant females in the United States have lower iron intake” (p. 1968).	Methodology: • Cross-sectional • No theoretical framework	Data collection: • NHANES 1999–2002 Data analysis: • Independent samples *t*-tests and χ^2^ • Geometric means Logistic regression analysis	Sampling strategy: • Oversampling Sample: • *n*=1045 females • Ages 13–54	Definition: See Laraia et al.^2^ Measurement tool: USFSS Cronbach α > 0.85 Outcome: Dichotomous	• Dietary iron intake not associated with food security status. • However, bioavailability to iron-rich foods differs among food secure and insecure people. Ferritin levels to diagnose iron deficiency • Food insecurity had higher prevalence, *p*=0.0025
Sandoval et al. (2021)^31^ Location: Los Angeles, California (West)	“Examines the association between clinically-identified prenatal household food insecurity and child premature gestational age and postnatal social needs” (p. 3).	Methodology: • Cross-sectional	Data collection: • EMR data extraction • Survey Data analysis: Logistic regression modeling (Poisson)	Sampling strategy: • Convenience Sample: • *n*=268 pregnant mothers • Low risk pregnancy	Definition: See Laraia et al.^2^ Measurement tool: USDA 6-item short form Outcome: Dichotomous	• Prenatal household good insecurity was associated with postnatal housing instability, legal needs, and transportation barriers. Food insecurity associated with higher odds of social needs OR 3.4 (1.5–8.0), *p*=0.004
Stevens (2010)^39^ Location: Washington State (Northwest)	“To explore the experience of food insecurity of young mothers and identify strategies used to manage food-insecure periods” (p. 163).	Methodology: • No clear methodology • No theoretical framework	Data collection: • Cognitive interview (semi-structure interviews) • Survey via USFSS (Cronbach's α > 0.85) Data analysis: • Thematic analysis Coding by Bickel and Nord	Sampling strategy: • Purposeful sampling • Snowball Sample: • *n*=19 • Young mothers 15–24 years old • Head of household • Parenting at least one child • English speaking	Definition: “Access by all members at all times to enough food for an active, healthy life. Food security includes at a minimum: (1) the ready availability of nutritionally adequate and safe foods; (2) assured ability to acquire acceptable foods in socially acceptable ways (this is, without resorting to emergency food supplies, scavenging, stealing, or other coping strategies” (p. 164). Measurement tool: USFSS Outcome: Categorical	• 76% (*n*=16) had food insecurity compared to the national average of 11% • 90% reported unstable housing Contributing factors for food insecurity • Affordable food sources • Unstable housing • Transportation • Income
Sullivan et al. (2021)^23^ Location: Cincinnati, Ohio (Midwest)	“To describe the food insecure population in an urban academic health center perinatal cohort” (p. 1).	Methodology: • Prospective cohort study • No theoretical framework	Data collection: • USDA Food Insecurity survey Data analysis: • Log binomial model Generalized linear model	Sampling strategy: • Convenience sampling Sample: • *n*=451 postpartum women • 53.0% non-Hispanic black women • 39.0% non-Hispanic white women • 4.0% other	Definition: “A household-level economic and social condition of limited or uncertain access to adequate food” (p. 1). Measurement tool: Modified version of USDA Adult Food Security Survey Module Outcome: Dichotomous	• One in five women were classified as food insecure. • Food insecure women were significantly more likely to experience a decrease in resilience. • Income is the most influential risk factor. Characteristics of study population • Housing instability *p*=0.002 • Smoking during pregnancy *p*=0.005 Adjusted risk factors associated with food insecurity • Annual household income < $40,000 Adj. RR 2.62 (1.18 to 5.84) • Obesity Adj. RR 1.69 (1.09 to 2.63) Descriptors of resilience • Feel little to no love • Moderate to extreme amount of despair • Less control over life *p*<0.001

BMI, body mass index; CFSM, Core Food Security Module; CI, confidence interval; DBP, diastolic blood pressure; EAT, eating attitude test; GDM, gestational diabetes mellitus; EMR, electronic medical record; NGDM, non gestational diabetes mellitus; OR, odds ratios; PIH, pregnancy-induced hypertension; PNC, prenatal care visits; RCT, randomized controlled trial; RR, relative risks; SBP, systolic blood pressure; SE, standard error; SNAP, Supplemental Nutrition Assistance Program; WIC, Women, Infants, and Children.

The following patterns were identified by pregnant and postpartum people who experience food insecurity: (1) psychological consequences, (2) physiological abnormalities, and (3) maternal hardships and inequities.

### Psychological consequences

Nine studies reported psychological consequences associated with food insecurity that might contribute to mental health crises during pregnancy and postpartum.^2,23–25,28,31,36,37,39^ The data revealed the following three subcategories: depression, anxiety and stress, and personal disposition issues.

#### Depression

In three studies, experiencing even marginal food insecurity was associated with developing symptoms of depression.^2,24,36^ Postpartum women with marginal food insecurity had 1.5 times the odds of having depressive symptoms compared with those in food secure households while controlling for income, race, education, marital status, and number of dependents.^2^ Feelings of hopelessness,^37^ despair,^23^ and lack of control^2^ contributed to depression while experiencing food insecurity. The Center for Epidemiological Studies Depression Scale, which is validated to assess for risk of depression in pregnant populations, was used in three of the studies;^2,24,37^ however, because symptoms of depression such as changes in appetite or fatigue can be nonpathological characteristics of pregnancy, assessing for depression was described as challenging.

#### Anxiety and stress

During pregnancy and postpartum, the main contributor to stress and anxiety was feelings of worry in having enough food for their families, particularly for other children in the home.^25,37,39^ Economic hardships, such as balancing monthly household finances, were considered an additional source of stress^31,39^ particularly among low-income communities, regardless of receiving public assistance such as Supplemental Nutrition Assistance Program (SNAP) or Women, Infants, and Children (WIC).^2,28,37,39^ In a study of food insecurity among adolescents, newly postpartum mothers had to decide whether to pay their rent or provide food for their families,^39^ contributing to overall stress.

#### Personal disposition issues and coping

Three articles described maladaptive behaviors that were associated with having food insecurity.^2,23,37^ Laraia et al.^2^ found that pregnant women experiencing household food insecurity developed a poor sense of self. Pregnant women from food insecure households in this study scored three times lower on the Rosenberg's Self-Esteem Scale used in this study compared with those from food secure households.^2^ For context, a low score indicates a less favorable attitude about oneself. There was also a development of loss of control over one's life or that life was controlled by chance.^2^ Sullivan et al.^23^ had similar findings; 22.4% of food insecure participants felt they had less control over their own life. Smoking, drinking, and illicit drug use were significantly correlated among pregnant people experiencing food insecurity.^23,37^ However, it is unclear from these two studies if participation in these activities during pregnancy was a result of food insecurity or other life factors.

### Physiological abnormalities

Fourteen articles identified some factors that might contribute to physiological changes in pregnant and postpartum people who experience food insecurity.^2,25,27–30,35,39^ Altered eating patterns and behaviors, gestational weight gain, pregnancy complications, and breastfeeding changes were identified as subcategories.

#### Altered eating patterns and behaviors

Minimal food intake or completely skipping meals was described as eating patterns among pregnant research participants.^23,25,27,39^ Nonhomeostatic eating, described as consuming calorie-dense, non-nutritious foods, was a common eating pattern for food insecure pregnant women.^2,23,27,39^ Laraia et al.^27^ found that eating attitudes and behaviors, such as restricting food due to concerns of weight gain, were poor during pregnancy and worsened postpartum in food insecure groups. The study reports a sensitivity analysis controlling a portion of the sample that falls at or below 400% of the federally poverty line and results remained statistically significant at the 0.05 level. This shows that there are additional barriers to obtaining nutritious foods that affect behaviors toward eating.

#### Gestational weight gain

Four studies examined gestational weight gain and food insecurity with conflicting results. In three studies, an excess in gestational weight gain, defined as a ratio calculated based on prepregnancy BMI,^16^ was found among food insecure groups.^27–29^ In contrast, Cheu et al.^25^ found insufficient weight gain and lower total gestational weight gain (*p*<0.001) associated with food insecurity. Notably, the three studies that found an excess in gestational weight gain were all secondary analysis from the same prospective cohort study.

#### Pregnancy complications

Some studies demonstrated associations between food insecurity and hypertension during pregnancy,^23,30^ second trimester anemia,^35^ and GDM.^26,29^ In an adjusted model used by Laraia et al.,^29^ the odds of GDM within a food insecure household were 2.38 times the odds of GDM within a food secure household (*p*<0.05). In a cohort study by Sullivan et al.,^23^ of the participants who had preeclampsia during pregnancy, 29.8% were food insecure compared with 18.4% of those who were food secure (*p*=0.02). The etiology of hypertension in pregnancy is complex; however, Morales et al.^30^ found that participation in a food resource program, which initiated SNAP, WIC, and access to a food pantry, improved overall blood pressure throughout pregnancy compared with those who did not participate.

#### Breastfeeding

Five studies addressed the effects of food insecurity on breastfeeding. Of these, two studies focused on infant health and breastfeeding as a childhood obesity prevention behavior.^32,40^ Four quantitative studies investigated the effect of food insecurity on breastfeeding initiation and continuation.^32–34,38^ Studies addressed additional child-specific outcomes, including restrictive and pressured feeding styles,^32^ or specific feeding behaviors such as early introduction of solid foods before 4 or 6 months of age^38^ or adding cereal to a baby bottle during feeds^32^ as a result of food insecurity. None of the included studies investigated positive maternal health outcomes associated with breastfeeding, such as postpartum weight loss, involution promotion, decreased postpartum bleeding, or improvements in glucose metabolism for mothers diagnosed with GDM.

Findings associating food insecurity with breastfeeding behaviors were mixed. One study found no significant association between food insecurity and current breastfeeding in a sample of WIC-eligible mother–infant dyads 2 weeks to 6 months postpartum.^32^ However, Frazier et al.^33^ found in adjusted analysis controlling for sociodemographic variables that food insecure mothers were significantly less likely to continue breastfeeding.

### Maternal hardships and health inequities

The prevalence of experiencing food insecurity was higher among low-income, single, African American and Hispanic pregnant people in their 20s.^2,25,30^ Income was an independent risk factor for food security concerns.^2,23,39^ Participation in federal government assistance programs, such as SNAP and WIC, ranged from 30% to 90%.^2,33,34,37,39,40^ Support from such programs was consistently described as insufficient to support food security.^39^ When parents decided to feed their infant formula, the assistance was also insufficient, as the monthly stipend decreased.^34^ Experiencing housing instability^23,30,36,39^ was not only associated with food insecurity but also made obtaining support from government assistance programs unattainable, as a permanent address may be required.^40^ Transportation challenges^31,39^ were also associated with food insecurity during pregnancy. Immigrant women noted the need to financially support family members in their home country as an added stressor that affected their ability to purchase food.^40^

Lastly, Laraia et al.^36^ found that experiencing marginal, low, and very low food security was significantly associated with lack of practical support and intimate partner violence.

### Common strengths and weaknesses

A main weakness among the included articles was a lack of explanation of screening tool modification to address household food security or insecurity in pregnancy.^23,25,30,36,37^ The studies with modified surveys did not report reliability statistics. The variability in definitions and measurements of food insecurity was a source of ambiguity in the data included in this review. One study described a theoretical framework utilized in the research.^36^ Clear objectives and significance were a common strength throughout the articles. Statistical analyses were appropriate for most studies.

## Discussion

Our findings suggest that pregnant and postpartum people experiencing food insecurity face greater hardship, and worse physiological and psychological outcomes than those who do not. Studies identified a mental health component that suggests food insecure pregnant people are at greater risk for developing depressive symptoms and anxiety. A cumulation of these adverse psychological factors can significantly impact mental health concerns during pregnancy and postpartum. The data suggest that clinical diagnoses of depression may be underreported in pregnant and postpartum people. A small body of evidence shows an association between food insecurity and metabolic complications of pregnancy. For example, Cooper et al.^26^ and Lairia et al.^2^ found an association between GDM and food insecurity. GDM is known as the most common metabolic disorder in pregnancy^17^ and can serve as a catalyst to poor maternal outcomes during birth,^42^ postpartum, and throughout the lifecourse.^43^

Definitions and measurements of food insecurity varied throughout the literature. For example, in 2006, Laraia et al.^2^ used the 18-item survey from the USDA, operationalized its outcome as food secure, marginal food security, and food insecurity, and defined food insecurity as “whenever the availability of nutritionally adequate and safe food, or the ability to acquire foods in a socially acceptable way is limited or uncertain” (p. 177). The most recently published study used a six-item survey that was modified from the original USDA survey, operationalized as fully food secure, marginally food secure, low food security, and very low food security, and described food insecurity as multidimensional and a health risk that has implications across the lifecourse.^36^

Lack of consensus on measuring and operationalizing food insecurity can lead to conflicting results in food security status. For example, “classifying” a household as marginally food secure may include questions that were answered negatively that would otherwise lead a household to be classified as food insecure. Understanding the rationale for the choice and adaptation of survey questions would contextualize the result of this review.

Regional differences might have contributed to some conflicting results in pregnancy outcomes described by Cheu et al.^25^ and Laraia et al.^27,29^ The discrepancy in gestational weight gain may be explained by differences in food environments, such as food deserts and food swamps. Food swamps are areas where fast food and convenience stores outnumber healthy food options and are identified in the included articles as contributing to excessive gestational weight gain as they are easily accessible and inexpensive.^27–29^

Health risks are created and maintained by faulty social systems^44^ influenced by structural racism and patriarchal forces. Food insecurity emerged as a result of the “Hunger in America” crisis starting in the 1960s, although federal government assistance programs had already existed.^45^ Federal government assistance programs were originally designed to be temporary “emergency programs” to support farm products.^46^ However, as economic conditions worsened, programs became permanent and ownership of small farm resources transitioned to agribusinesses.^46^ The USDA is the government agency that provides support for capitalized farms in addition to food and nutrition programs, such as SNAP and WIC.

SNAP and WIC also rely on the USDA for nutrition guidelines and advice; specifically, the Food and Nutrition Service Agency (FNS).^47^ FNS's mission is to “increase food security and hunger in partnership with cooperating organizations by providing children and low-income people access to food, a healthy diet, and nutrition education in a manner that supports *American Agriculture* and inspires public confidence.”^3^ However, The Farm Bill, which provides the safety net for SNAP, also serves as the safety net for farmers.^47^ While revisions of the Farm Bill have taken place since its initiation,^47^ the highest subsidy is primarily for commodity crops, such as corn, wheat, and soy, which are basic ingredients in processed foods^46^ and show an association with cardiometabolic conditions in Americans.^48^

Although there are benefits to SNAP, there are pitfalls. SNAP relies on the Thrifty Food Plan, one of the four plans designed by the USDA to be nutritionally adequate at a low cost using a benefits formula.^49^ The formula operates on assumptions of household expenditures, of which 30% is allocated for food. It does not consider time (food preparation), equipment (food utensils and supplies), and knowledge of its recipients. It also does not consider price variation, which can be complicated by geography or disruptions to the supply chain. Pregnant individuals are not eligible for increases in SNAP until after the neonate is born.^2^ It should be noted that WIC relies on a separate funding source and receives significantly less than SNAP.^47^ This history potentially supports findings from Stevens that monthly disbursements are insufficient.^39^

Studies in this review reported that their samples comprised women, with no data provided on whether more detailed aspects of gender identity were collected. Further exploration of the role of food insecurity among people across the gender spectrum who have the capacity for pregnancy is needed. Only one study included acculturation^37^ as a sociodemographic characteristic to describe food insecurity. Therefore, there are likely cultural and ethnic considerations to what food insecurity means to different groups.

## Implications

### Policy

Food insecurity persisted when controlling for public assistance use along with sociodemographic indicators demonstrating that current federal assistance programs do not go far enough in alleviating the burden of food insecurity in pregnant and postpartum people. Given recent inflation due to the COVID-19 pandemic,^10^ federal, state, and local governments must reevaluate their nutrition and housing expenditures to ensure that they are doing enough to support recipients, particularly pregnant and postpartum recipients. In addition, lawmakers should consider the pitfalls of the assumptions that household expenditure on food is only 30%. This could potentially mitigate the financial inequities that contribute to food insecurity among pregnant and postpartum people.^23,30,39^ Finally, inflation on fresh fruits and vegetables compared with the price of processed foods is directly related to farm policies within the USDA.^46^ Subsidies for fresh fruit and vegetables should be higher than those for soy, wheat, and other commodity crops.

### Health services utilization

Screening for household food security at least once during pregnancy is recommended by the American College of Obstetricians and Gynecologists and the American Academy of Pediatrics.^50^ The USFSS tool asks a series of questions pertaining to food security within the last 12 months. Food insecurity waxes and wanes at different times,^24^ and therefore asking at one point in time may not identify all potential risks when considering the trajectory of pregnancy, postpartum, and beyond.^24^

A possible solution could be to routinely assess food security at prenatal and postpartum visits. However, none of the studies discussed the responsibility of the obstetrical team to screen. Only one study had an established prenatal social needs screening and risk assessment program with identified pathways for social work and care coordination referrals.^31^ Postpartum social needs were identified in pediatric settings.^31,39^ In pediatric settings, mothers experienced embarrassment and shame when discussing food concerns.^39^ Poor patient experiences suggest that regular conversations on food insecurity screening could decrease the stigma felt by pregnant and postpartum people and that conversations should occur early in prenatal care, so that they are well-received later in pregnancy and postpartum. Therefore, screening for food insecurity and other SDOHs should be included in primary care settings for all people with the capacity of pregnancy so that appropriate referrals to either government or community programs can be made.

A greater effort needs to be made to meet people where they are in the community to address their food-related needs, for example, a partnership with community-based food gardens^51^ that can receive referrals for food insecure individuals and supply fruits and vegetables to those who need it.

### Research

Future research should focus on the association between built neighborhood environment, maternal health outcomes, and the lived experiences of pregnant and postpartum women who experience food insecurity. An explanatory mixed-methods design could provide contextual support of barriers to achieving food security and postpartum health. Research of this nature could support the need for policy reform of public assistance programs. Longitudinal studies would explain how food insecurity affects the life span and establish temporal and causal relationships between food insecurity and health outcomes. For example, evaluating the multigenerational impact of breastfeeding behaviors, food insecurity, and maternal health outcomes, in studies that assess food security and its association with breastfeeding behaviors where research could uncover multigenerational findings.

### Limitations and strengths

This integrative review has several limitations. First, we only included studies published in the United States and written in English, which limited the overall number of eligible studies. Five of 19 articles used data from the same observational cohort study, with the same primary author, which could have skewed results. It is unclear if rural settings are represented in this review, and therefore, the results may not be generalizable. In addition, due to the scarce representation of Alaska Native/Pacific Islander and native/indigenous participants, this review cannot speak to their unique strengths and adversities. Strengths include two independent data abstractors, support from a health services librarian, and use of critical appraisal.

## Conclusion

An interdisciplinary approach to addressing food insecurity in pregnant and postpartum people has the potential to change our communities for women and people of reproductive age. As demonstrated in this review, the significance of food insecurity in pregnancy is multifaceted and should be considered a critical opportunity to address issues in one's overall health. Short-term solutions involve screening and referrals to community or federal government programs. Long-term solutions include addressing the root causes of food insecurity, such as accessibility and affordability, which requires a collective effort from policy makers, health care providers, and community programs. If not addressed, there are potentially long-term threats to maternal health, both reproductive and beyond.

## Abbreviations Used

BMI: body mass index
CASP: Critical Appraisal Skills Program
CFSM: Core Food Security Module
CI: confidence interval
DBP: diastolic blood pressure
EAT: eating attitude test
EMR: electronic medical record
FNS: Food and Nutrition Service Agency
GDM: gestational diabetes mellitus
NGDM: non gestational diabetes mellitus
NHANES: National Health and Nutrition Examination Survey
OR: odds ratios
PIH: pregnancy-induced hypertension
PNC: prenatal care visits
RCT: randomized controlled trial
RR: relative risks
SBP: systolic blood pressure
SDOHs: social determinants of health
SE: standard error
SNAP: Supplemental Nutrition Assistance Program
USDA: United States Department of Agriculture
USFSS: U.S. Household Food Security Survey
WIC: Women, Infants, and Children
